# Expanding Access to Essential Quality Services for Cancer Patients as Part of Universal Health Coverage: Reflections From the World Cancer Leaders’ Summit

**DOI:** 10.1200/GO.20.00020

**Published:** 2020-03-26

**Authors:** Sonali Elizabeth Johnson, Rowena Tasker, Dina Mired, Anil D’Cruz, Cary Adams

**Affiliations:** ^1^ Union for International Cancer Control, Geneva, Switzerland

## INTRODUCTION

In October 2019, more than 300 participants from 80 countries came together for the World Cancer Leaders’ Summit, the theme of which was Cancer and Universal Health Coverage. The summit was cohosted by the Union for International Cancer Control, WHO, the International Agency for Research on Cancer (IARC), and the International Atomic Energy Agency. Attendees included policy makers and representatives from international agencies, non-governmental organizations, academia, hospitals, and industry. This was the eleventh in a series of summits that began in 2008.

Universal health coverage (UHC) has often been described as a political choice, including by the Director General of WHO, Tedros Ghebreyesus, PhD.^1^ Consensus around the need for UHC resulted in the first United Nations High-Level Meeting in New York in September 2019. The meeting saw the adoption of the Political Declaration of 2019, “Universal Health Coverage: Moving Together to Build a Healthier World,” which sets out a high-level framework for the development and implementation of national UHC plans. The World Cancer Leaders’ Summit on Cancer and UHC came less than a month after this landmark meeting. The Summit was a timely opportunity to discuss how the cancer community can ensure that cancer interventions are included in UHC benefits packages as they are rolled out in low- and middle-income countries (LMICs) and how quality cancer care can be sustained in high-income countries against the backdrop of rising health care costs.

The summit was focused on the overarching political economy of UHC and the opportunities for cancer advocacy. The question was asked: How do we expand access to essential, quality services for cancer patients worldwide as part of the global drive to achieve UHC? The discussions looked at ways to leverage national health policy to strengthen care for patients with cancer and noncommunicable diseases (NCDs) more broadly, focusing on the national structures needed to reframe health care governance and planning. We discuss three themes that emerged as key drivers for stronger action for controlling cancer within UHC:

1. Increasing levels of health financing
2. Building strong health systems
3. Improving health equity.

### Increasing Levels of Health Financing

Around 170 million healthy life-years are lost because of disability and death from cancer,^2^ with an estimated annual cost of approximately US$1.2 trillion which increases to US$2.5 trillion when factoring in the additional costs of untreated pain and suffering.^3^ Consequently, the need to ensure adequate and sustainable financing for cancer control is essential to achieving UHC. Recent studies demonstrate that current financing for cancer is insufficient, and this places significant health and economic costs on patients and their families who pay out-of-pocket for cancer treatment and care.^4^

Given that protection from financial catastrophe is one of the three central tenets of UHC, implementing UHC programs nationally will require far greater engagement from Ministries of Finance and other government sectors than has previously been seen in health policy. Although the need for a whole-of-government response to health has long been called for within global health dialogues, efforts to achieve UHC emphasize both health and financial outcomes as metrics of success. It is therefore encouraging that governments are beginning to evaluate the impact of current health financing mechanisms on patients’ ability to access health services and on levels of out-of-pocket health spending.

In Kazakhstan, a national commitment by the president to deliver UHC helped stimulate a new collaboration between the Ministry of Finance and the Ministry of Health. The starting point was a comprehensive national review of financing for health, which found that national investment stood at 3.6% of gross domestic product, whereas out-of-pocket spending was at 38%.^5^ In response, the Ministries are working together to establish a mandatory social insurance fund to support UHC that will complement the existing national essential care package. It is anticipated that the development of this fund will liberate some government investments to strengthen the foundations of the health system, with a commitment to increase public health spending to 5% of gross domestic product.

The development of new funding models to complement existing investments in health was a common theme throughout the discussions, mainly related to fiscal policy and sin taxes, with examples from Uruguay and Mongolia which leverage tax on alcohol and tobacco. In addition, Uruguay taxes sugar-sweetened beverages to develop a catalytic fund for UHC investments. Public-private partnerships are also being used to mobilize additional resources, and as these partnerships grow, governments will need to develop robust regulatory frameworks to ensure the quality of services.

Although securing additional funds to support the prevention and control of NCDs is essential, participants emphasized that funds must be used as efficiently as possible. To enable smoother and more systematic collaborations with the Ministry of Finance, cancer planners within the Ministry of Health need to demonstrate the return on investment and feasibility of implementing and sustaining essential cancer services within the country’s health system. Although we lack reliable estimates on global public health spending on cancer, we know that the costs of improperly or inadequately treated cancer exceed US$895 billion, clearly demonstrating the opportunity we have to make timely and effective investments in cancer care that can curb the burden of cancer and help all countries advance toward the global UHC goal.^6^ There is an increasing body of evidence to support the case for investment in priority cancer services to underpin advocacy in cancer control. For example, it is estimated that an essential package of cost-effective measures for cancer that includes tobacco control; prevention of cancer-causing infections; diagnosis and treatment of breast, cervical, and selected childhood cancers; and provision of readily available palliative care would constitute only 3% of total public spending on health in LMICs.^7^ The forthcoming WHO/IARC costing tool for cancer interventions was presented at the Summit and will be an important source of guidance for countries on planning and resourcing cancer control. The costing tool (based on the One Health Tool approach) will identify cost-effective interventions according to the country context, develop a resource-stratified approach that accommodates different health system capacities, and support the setting of priorities.

### Building Strong Health Systems

The drive to achieve UHC cannot be accomplished without strengthening the capacity of health systems to respond to the needs of patients. To this end, there has been a strong focus on adopting a robust primary health care approach as the primary vessel through which countries can improve equitable access to health services.

Early detection and diagnosis of cancer reduces the burden of cancer on patients, their families, and health systems. A cervical cancer elimination model presented at the Summit demonstrated that achieving the targets of 1) 90% of girls being fully vaccinated against human papillomavirus by 15 years of age, 2) 70% of women being screened with a high-precision test at 35 and 45 years of age, and 3) 90% of women being identified with cervical disease receiving treatment and care—known as the 90-70-90 targets—would result in the elimination of cervical cancer in a century and make significant strides toward meeting the overall reduction in premature mortality as a result of cancer by 2030, in line with Sustainable Development Goal 3.4 (SDG 3.4).

Although population-based cancer screening efforts for cancer sites such as bowel and breast require a health system capacity that is currently not available in most LMICs, early diagnosis of solid tumors followed by prompt treatment can be progressively realized as health systems are strengthened. This would include raising awareness about cancer in the general population, empowering people to seek medical attention promptly for suspicious signs and symptoms, and strengthening health worker training at the primary health care (PHC) level to identify early warning signs and set up referral protocols to secondary and tertiary services for appropriate management and follow-up.

Indeed, health promotion using primary care as the main platform was emphasized by participants as being a missed opportunity for cancer control. In Oman, mobile units are used in rural areas to raise awareness and screen eligible women for breast cancer to help tackle the issue of late diagnosis, and doctors now report diagnosing cancer at a much earlier stage than before this service was introduced. Health promotion activities to raise awareness of cancer have also been used in other high-income countries, including Canada and Australia, where screening uptake is low among some populations. Uruguay also highlighted the importance of creating an enabling environment for health that involves pairing tobacco control laws with greater information on smoking cessation and other prevention activities to effectively respond to the growing burden of cancer associated with alcohol use and being overweight or obese.

Although PHC is an important part of developing a stronger health system, the care pathway for cancer cannot be oversimplified and compared with that for other diseases such as HIV/AIDS and tuberculosis that are predominantly detected and managed at the PHC level. The model of cancer management will differ from the traditional communicable disease responses, which tend to focus on behavioral change, information dissemination, and access to a single regimen. Many of the participants and speakers commented on the unique challenges they face in reducing the cancer burden, given the need for a comprehensive approach that engages all levels of the health system and requires specialists in oncology. The drive to deliver patient-centered care with robust referrals and support for individuals between levels of the health system is critical for the successful treatment of cancer, most notably in reducing delays between diagnosis and treatment. However, adopting an integrated cancer management approach would have benefits beyond cancer to other NCDs and would help respond to the increasing number of patients with multiple morbidities.

### Improving Health Equity

Inequities in cancer care and in cancer outcomes are found globally, irrespective of income level. In Europe, the WHO regional office shared how cancer outcomes vary widely across the region, in which less privileged groups have worse outcomes because of a combination of higher exposure to risk factors and less access to screening programs and health services overall; those groups are also more profoundly affected by the social and financial costs of cancer. Low health literacy among populations in the region is associated with worse health outcomes, more hospitalizations, lower uptake of screening and preventive services, and poorer overall health.^8^ Consequently, WHO is working with European countries on the socioeconomic aspects of care and launching a new multi-stakeholder drive to improve health literacy. Much of this initiative will take place at the PHC level and will adopt a life course approach to ensure that individuals at different stages in their life cycle receive the information and services they require.

The challenges in reaching rural or underserved populations with cancer services were discussed by several delegates. A unique case study presented from Mongolia, a country that is geographically large and has a low population density. With temperatures ranging from –40°C to 40°C and with populations living in inaccessible locations, health workers frequently demonstrate flexibility and ingenuity in transporting medicines and supplies, often traveling by horse and reindeer to access remote populations. Mongolia’s health system has invested in the expansion of mobile clinics to deliver basic health interventions. Cancer has been included in this effort with the introduction of breast and cervical cancer screening in 2012 and screening for liver cancer in 2016.

Addressing the problem of inequities among cancer patients underscores the role of civil society organizations in bringing the issue of health disparities linked to ethnicity, gender, wealth, and other social inequalities to light through research and advocacy as well as working with governments to design evidence-informed policy responses. One innovative session at the Summit (presented by the McCabe Centre for Law and Cancer) assessed the effective use of the law to achieve stronger cancer control. Examples were provided from countries such as Mexico, where the Cancer Warriors of Mexico successfully pushed a bill through the Mexican Congress to grant occupational leave to parents whose children are undergoing cancer treatment. Participants learned how, in many instances, the law can be a strong enabler for UHC. However, when laws are poorly designed, legislation can lead to barriers in accessing care and further entrench inequities. Consequently, it is essential to understand the role of legislation in health governance and carefully assess whether proposed laws and policies can alleviate or promulgate discrimination or inequity under UHC.

In conclusion, the momentum for UHC around the world provides the cancer community with important opportunities for engagement. The key mechanism will be aligning the priorities in national cancer control plans with national UHC plans. It will be important for all actors, particularly civil society organizations, to increase national and regional advocacy in the areas of increasing health financing, developing stronger health systems that can support the management of NCDs, and addressing inequities to ensure that all groups, including patients with cancer, benefit from UHC. In the final call to action, the Union for International Cancer Control emphasized that we are not starting from scratch. Rather, we already have existing commitments to advance the control of cancer, including the 2017 World Health Assembly Cancer Resolution, the Global Action Plan on NCDs, and the United Nations Political Declarations on NCDs and UHC. As we begin a new decade, it is time to follow through on these.
